# Does minimally invasive percutaneous transilial internal fixator became an effective option for sacral fractures? A prospective study with novel implantation technique

**DOI:** 10.1007/s00068-022-02212-6

**Published:** 2023-01-24

**Authors:** Elsayed Kassem, Sherif A. Khaled, Mahmoud Abdel Karim, Ahmed Goda El-Hamalawy, Mahmoud Fahmy

**Affiliations:** Pelvis Fractures and Arthroplasty Unit, Cairo University, Cairo, Egypt

**Keywords:** Percutaneous fixation, Sacral fractures, Transilial, Internal fixator

## Abstract

**Aim:**

To assess radiological and functional outcomes of transilial internal fixator (TIFI) for treatment of sacral complete transforaminal fractures with a novel implantation technique that decrease wound irritation problems in addition to facilitating easy application of reduction methods beside showing the best entry points, screw trajectories and angles.

**Methods:**

A Prospective case series from 2019 to 2021 was conducted at university hospital including 72 patients with Denis type 2 sacral fractures. The operative and fluoroscopy time, reduction, implantation techniques, postoperative radiological and functional data were collected and evaluated with minimum follow-up of 12 months.

**Results:**

The mean initial fracture displacement was 4.42 mm while mean postoperative maximum residual fracture displacement was 2.8 mm, Radiological outcome assessed using Matta’s grading at the final follow-up visit with 63 cases scored as Excellent,7 cases as Good, 2 cases as fair. Functional outcome using Majeed scoring shows 64 cases of Excellent grading and 8 cases were Good. Short operative and fluoroscopy time, easy reduction techniques, few skin problems were recorded.

**Conclusion:**

TIFI through a minimally invasive technique represents a valid method for dealing with transforaminal sacral fractures. TIFI provides a rigid fixation for posterior ring injuries with few risks regarding iatrogenic nerve injury, avoiding different variations of upper sacral osseous anatomy or sacral dysmorphism. In addition, there is no necessity for high quality fluoroscopy for visualization of sacral foramina intraoperatively, decreasing risk of radiation exposure, unlike other methods of fixation as iliosacral screws. Our novel modification for implantation technique provides few risks for postoperative and wound complications.

## Introduction

Several posterior fixation methods have been reported for pelvic ring fractures, including ilio-sacral screw (IS) fixation, plate fixation, and spinopelvic fixation. Although IS screw fixation is minimally invasive, reduction is challenging, fixation may not be secure, and may cause nerve injury through fracture compression especially in transforaminal fractures [1, 2]. Moreover, upper sacral segment dysmorphism is observed with an incidence of 30–54% [3]. Transiliac plate fixation, considered to be an aggressive invasive technique for the posterior pelvic soft tissues. Spinopelvic fixation represents one of the most secure fixations for the pelvic ring. However, it is a lengthy invasive procedure compromising movement of the intact lower lumbar vertebrae. Transiliac internal fixator using a minimal incision for inserting only two pedicle screws and connecting them subfascial with a rod provides excellent biomechanical strength [1, 4–6].

Decision making can be made regarding suitable management depending on instability and displacement aiming for fracture union and physiologic orientation [7]. For any implant attached to the axial bones, a portion of the fixation tool should pass anterior to the pivot point which is demarcated as the centre of the bony column at the junction between L5 and S1[8–10].

Our aim was to assess radiological and functional outcomes of TIFI with a novel implantation technique and easy applicable reduction methods in addition to recording operative time and blood loss. The ideal entry points, screw trajectories and angles were studied to decrease radiation exposure and allow for the precise TIFI placement.

## Patients and methods

From January 2019 to December 2021, a case series was conducted in a level I trauma centre in a university hospital after obtaining approval from our institution’s ethical committee (MD-168–2020) to evaluate the results of the fixation of complete transforaminal sacral fractures with TIFI using a minimally invasive technique. 72 patients above 16 years old with transforaminal (even in comminuted or bilateral) complete sacrum fractures were included. Neglected, pathological and other types of sacral fractures were excluded. Posterior ring was fixed either alone or adjunct to anterior pelvic ring fixation according to pelvic fracture configuration and the preoperative planning. All patients were preoperatively evaluated clinically and radiologically, consented, and adequately prepared for the operation. Preoperative data including age, sex, mode of trauma, associated fractures, fracture classification and initial fracture displacement were collected. All fractures were classified using Tile’s classification and Denis classification. Sacral fracture displacement was assessed using preoperative plain X-rays (AP, inlet, outlet) with magnification 100% and reassessed using preoperative CT (axial, coronal, sagittal and 3D) quantifying the most displacement in any view in mm according to Matta measuring criteria (in maximum displaced view).

**Fig. 1:**
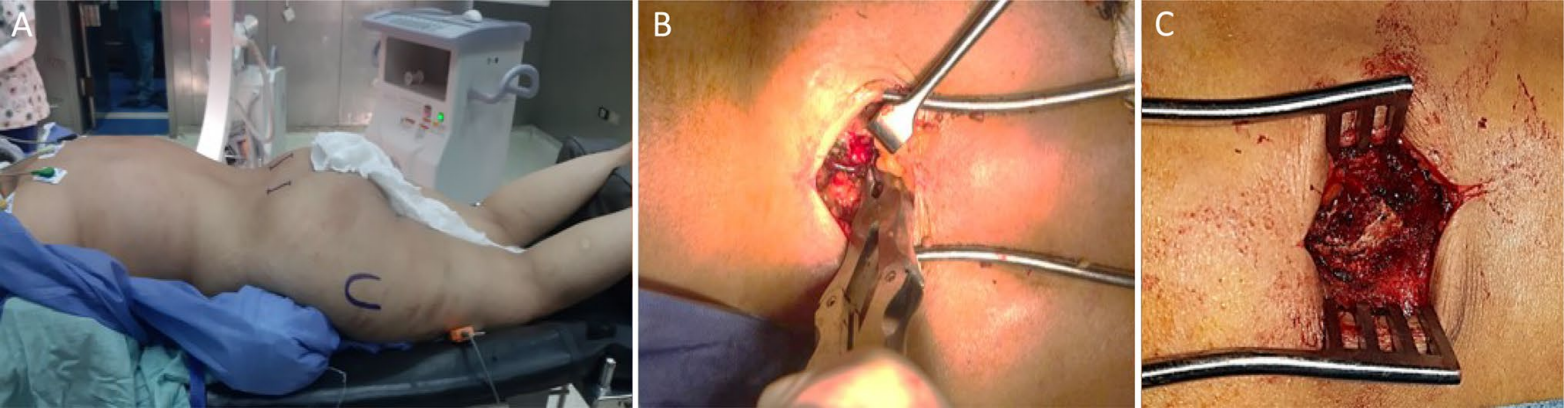
**A** Prone position on radiolucent table, marking of PSIS and greater trochanter on each side. **B** PSIS exposure: starting point located caudal and medial to PSIS (Anatomic entry point). **C** Rongeur bite used on the medial aspect of PSIS to reach the cancellous bone

**Fig. 2:**
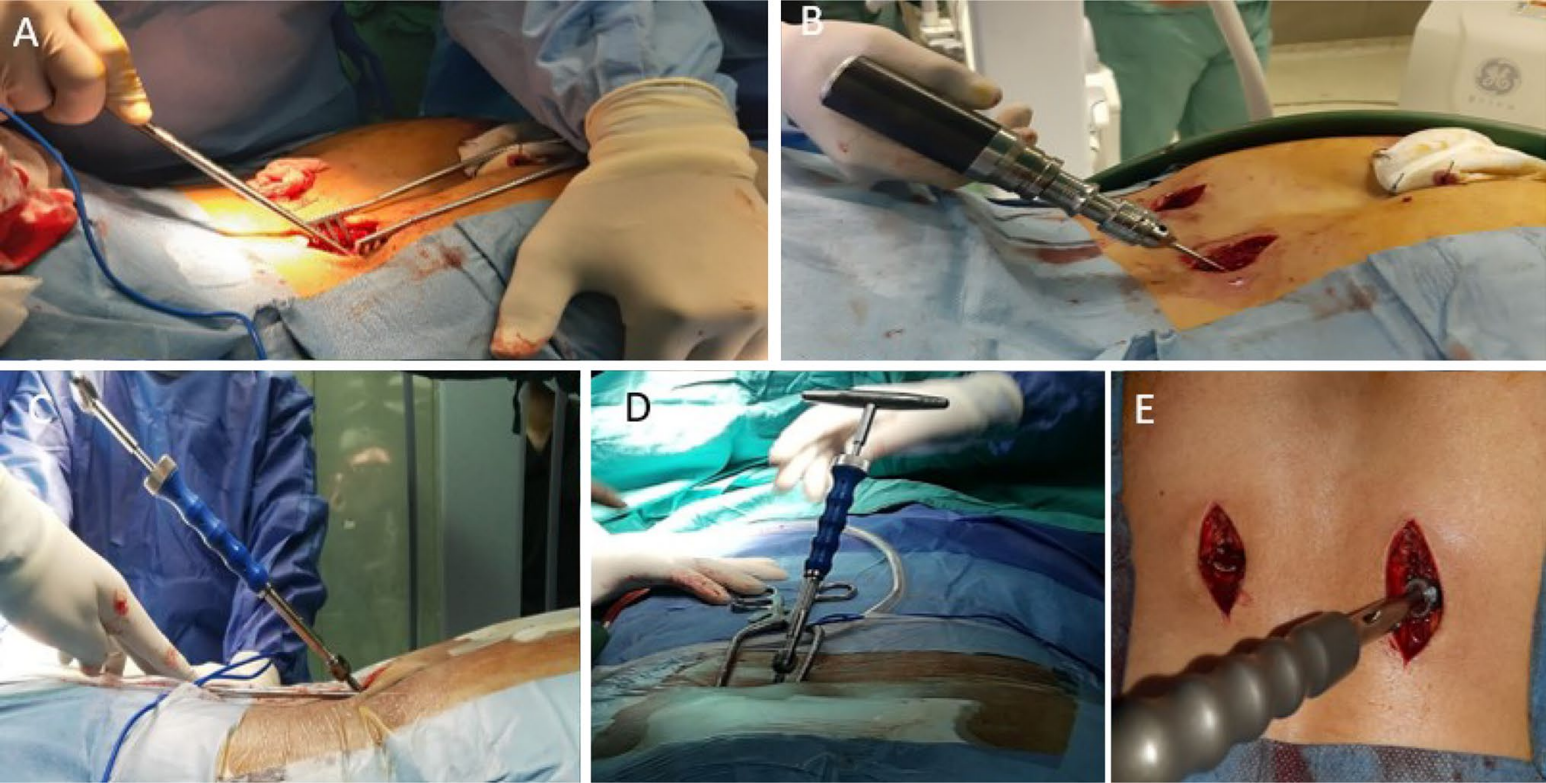
**A** Awl used for penetration and creating screw tunnel. **B** Alternatively, oscillating drill could be used instead. **C**, **D** direction of awl in ventral and caudal direction. **E** Pedicular screw insertion directed caudal, ventral towards greater trochanter aiming towards AIIS, then connection bar inserted submuscular and fixed to the pedicle screws

**Fig. 3:**
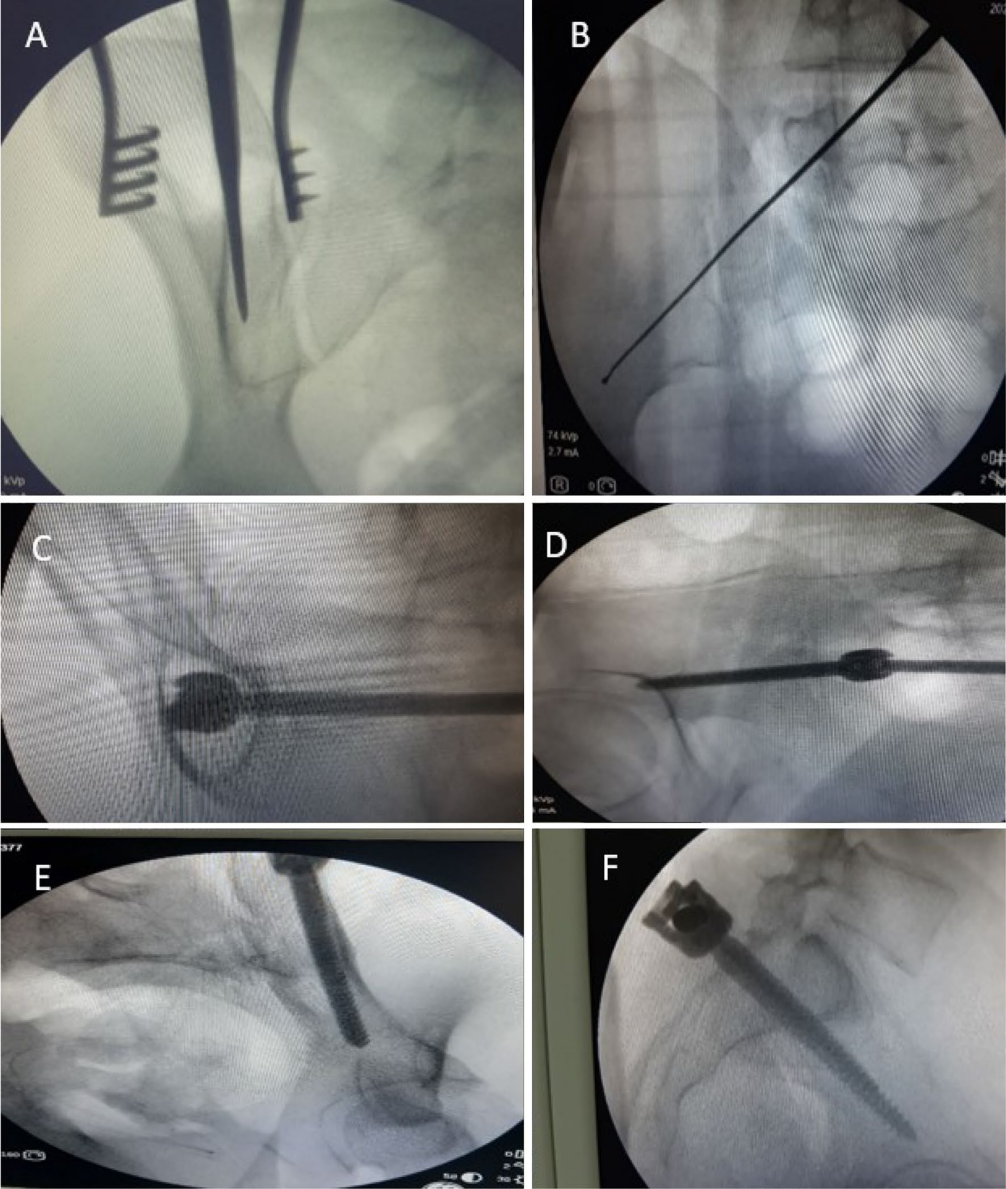
**A** Showing Obturator view with awl insertion between the 2 tables. **B** Screw placement above sciatic notch in iliac view. **C** Tear drop view, **D** Iliac outlet view. **E** Obturator inlet. **F** True lateral view

All surgeries were performed by one of the authors whom are senior pelvis and acetabular fracture consultants. Prone position for patients was done on a radiolucent table under spinal epidural anesthesia after preoperative prophylactic antibiotics (Fig. 1).We used a new implantation method for the iliac screws, starting first with 2 separate vertical incisions (2–4 cm) that were made just medial to the posterior superior iliac spine PSIS (one on each side) then the anatomical entry point that is just medial and caudal to the PSIS was determined after resecting bony prominence to decrease implant irritation (Fig. 2). A pedicular screw on each side (60–110 mm length and 6.5–7.5 mm in diameter) was inserted and directed towards the ipsilateral AIIS (Fig. 3). Finally, a single rod was applied submuscular and adequately reattaching the fascial flap and the muscle layer over the screw.

Fluoroscopic views were checked to confirm screw placement accuracy,trajectory,safe corridors, and length of iliac screws.Iliac,obturator, both obliques, combined views as tear drop view (which shows bony channel above the sciatic notch within which the screw should be positioned), iliac outlet view (which insures screw direction away from the hip joint), obturator inlet view (where the screw should remain within inner and outer margins of the ilium allowing visualization of its entire length from ASIS to PSIS) were done. Finally, a true lateral view was taken which is the primary view for iliac screw insertion (showing the screw trajectory that should extend within 2 cm above the sciatic notch, above the acetabulum and towards the AIIS) (Fig. 3).

**Fig. 4:**
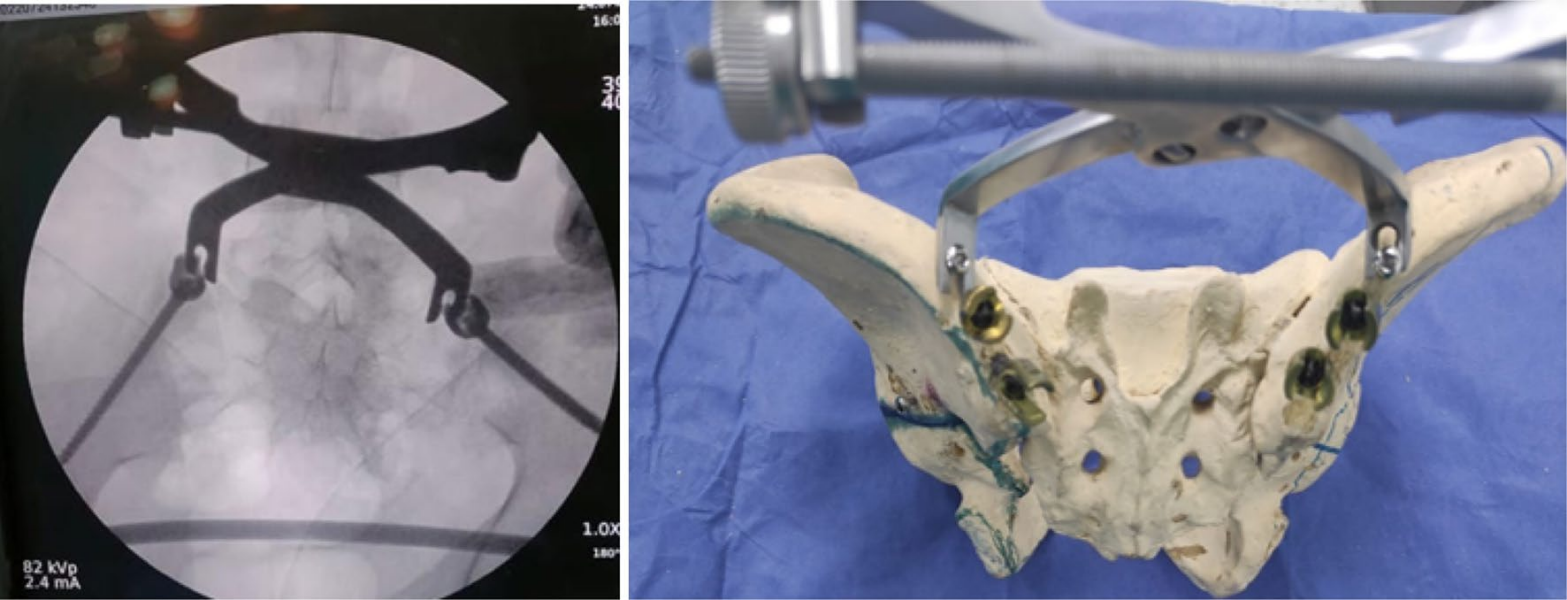
Fracture reduction methods using pelvic reduction clamps applied over 4.5 cortical screws

Proper closed reduction was done in all cases using large pelvic reduction clamp that applied firmly either to a reduction 4.5 cortical screws (that were applied temporarily above the planned pedicle screw area) or to the neck of a reduction pedicle screws that could be applied above the definitive applied pedicle screws. Using this clamp, vertical displacement is corrected together with applying a compression force over the fracture side while the dorsal displacement can be buttressed using a well contoured rod (Fig. 4).

**Fig. 5:**
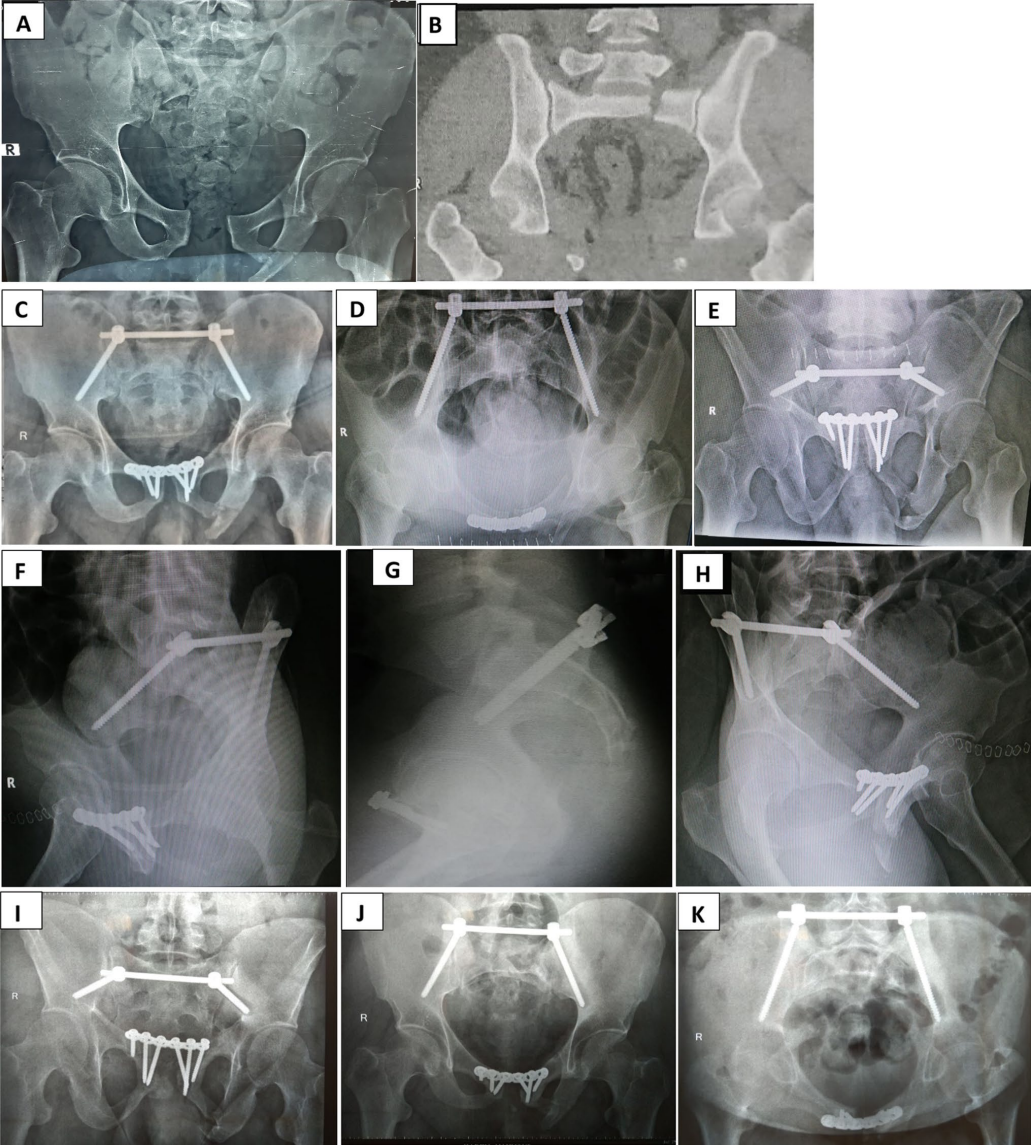
**A** Preoperative radiograph showing left fracture sacrum Denis type 2 in 40 years old male. **B**–**H** Postoperative radiographic views showing iliac screws accurate trajectory. **I**–**K** Final follow-up radiographs

**Fig. 6:**
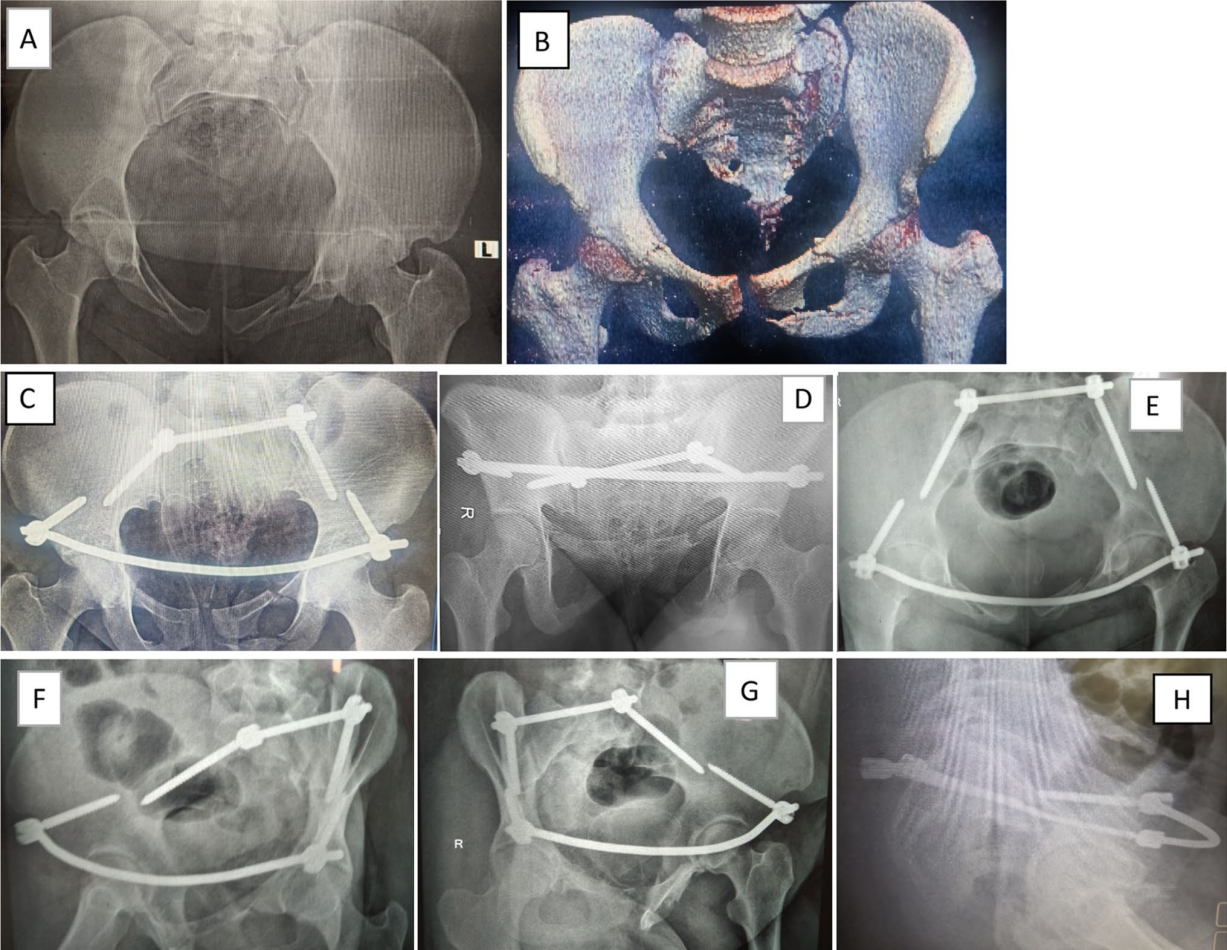
**A**, **B** Preoperative radiograph showing left fracture sacrum Denis type 2 in 21 years old male. **C**–**H** Final follow-up different radiographs showing iliac screws accurate trajectory

Regarding methods of management for anterior ring injury, examination under anesthesia was done for lateral compression cases to assess the stability of anterior ring after posterior fixation and 8 cases did not need anterior fixation (Figs. 5 and 6 show case examples).

Operative time, fluoroscopy time and blood loss, intra and postoperative complications were recorded and patients were followed up for a minimum of 12 months functionally using Majeed score, pelvic outcome score and radiologically using Matta score showing any residual fracture displacement. Healing of sacral fractures is very difficult to confirm on the basis of plane radiographs alone so sacral fracture union was assessed clinically (full weight bearing ability and disappearance of bony tenderness) and radiologically using Matta’s radiological outcome grading using plain x rays, every 2 months till the final follow-up visit.

All patients were allowed to mobilize in bed immediately postoperatively with knee and ankle exercises. Postoperative weight bearing depends on the stability of the anterior and posterior pelvic ring fixation, the quality of the patient's bone and the presence of associated injuries. Generally partial weight bearing was allowed after 2 weeks till the sixth week then fracture union was assessed clinically and radiologically to shift for full weight bearing.

Data were coded and entered using the statistical package for the Social Sciences (SPSS) version 26 (IBM Corp., Armonk, NY, USA). Comparisons between quantitative variables were done using the non-parametric Kruskal–Wallis and Mann–Whitney tests.

## Results

During the duration of our study, we received 198 cases with fracture pelvis, 111 cases had a fracture sacrum as apart of pelvic fracture. 10 cases were excluded due to age limit while 11 neglected cases were also omitted. 25 cases needed spinopelvic fixation, 10 cases showed nondisplaced incomplete fracture sacrum, 3 cases were Denis type 1 and all was excluded. Only 72 cases with complete longitudinal transforaminal fracture sacrum met our inclusion criteria.

The mean age was 30.3 ± 8.7 years (range 18–43 years). The mode of trauma was road traffic accident (RTA) in 35 patients, falling from height (FFH) in 20 patients, motor bike accidents (MBA) in 11 patients and motor car accident in 6 patients. The mean time between the date of trauma and surgery was 8.4 days (range 2–21 days). Regarding Tile classification, fractures were classified as: B1 in 9, B2 in 52, B3 in 8 and C1 in 3 cases, while according to Denis classification, all fractures were of Denis II type. The mean operative time was 38.9 min (range 22–65 min). The mean estimated blood was 312 ml (range 150–670 ml). the mean fluoroscopy time needed intraoperatively was 25.15 s ± 10.4

Regarding implants, single rod fixation was used in all patients, with an average pedicular screw length of 80 mm ± 9.5 (range 65–110 mm) and mean diameter of 7 mm ± 0.2 (range 6.5–7.5 mm).

37 cases were fixed in situ with no reduction, the other 35 cases were reduced using the reduction methods explained before and fixed percutaneously. The mean initial fracture displacement was 4.42 ± 2.92 mm (range 1–18 mm) while the mean postoperative maximum residual fracture displacement was 2.8 ± 1.57 (range 1–6 mm). Radiological outcome was measured based upon Matta’s radiological outcome grading at the final follow-up visit with 63 cases scored as (Excellent),7 cases as (Good) with 2 cases as (fair) with no poor results. In comparing initial displacement preoperatively and Matta Radiological outcome at 6 months, there was a statistically significant difference (*P*: 0.001) indicating that preoperative initial displacement affects Matta score.

The functional outcome, which was assessed using the Majeed score, showed 58 cases with good grade,12 with fair grade, 2 with poor grade and the mean was 68.5 ± 7.8 (range 58–82) at 6 months. 64 cases were excellent and 8 cases were good with a mean of 88.3 ± 4.7 (range 81–97) at the final follow-up. Mean Pelvic outcome score at 1-year follow-up was 33.5 ± 2.6 (range 29–37). Patients with associated fractures and poly traumatized patients showed lower functional outcome scores.

All cases showed sacral fracture union with a mean time to union of 3.8 months ± 1.2 (range 2–7 months). The mean follow-up period was 18.1 months ± 1.6 (range 12–26 months).

The relation between age of patients and time needed for union was of no statistically significant difference as well as the relation between age and functional outcome score that was also of no statistically significant difference at 1-year follow-up.

Anterior ring was fixed by fixing the anterior spinal system in 51 patients, symphyseal plate in 12 patient and external fixators in one case. No complications were encountered anteriorly with no effect on implant loosening or failure (either anterior or posterior) in all cases at the final follow-up visit.

4 cases showed posterior wound complications, 2 cases improved by repeated dressing and antibiotics, one case needed debridement and retention of implant while the other case showed late infection after 9 months with full union and good functional outcome so debridement and removal of implant was done. 4 cases had preoperative nerve injury due to transforaminal injury affecting L5, S1 nerve roots. All were improved within 3–6 months without the need for decompression. No iatrogenic nerve injury was reported from our technique.

## Discussion

Sacral fractures are complex challenging fractures that need to be reduced and fixed properly to restore its anatomy and mechanics [1, 11, 12]. Multiple treatment modalities exist, each with its own advantages and disadvantages. The experience of the operating surgeon is also a factor [13–15]. Fixation techniques remain a subject of argument regarding superiority of one fixation method over another [1, 16, 17]. Iliosacral (ISS) screw fixation is the most common method used being minimally invasive and of good biomechanical performance. However, hazards of neurological affection with radiation exposure are still a drawback, that was overcomed recently using the navigation technology [2, 3].

Sacral plating was used as an option for good exposure, open reduction and fixation but a major soft tissue problem was a limiting factor [3, 7, 9]. Lumbopelvic fixation or triangular osteosynthesis were recommended for unstable pelvic ring fractures as it is the most stable construct that resists high loads and stresses. However, wound complications are a major concern [3, 16, 17]. Recent literature stated that complete transforaminal longitudinal sacral fractures are considered to be unstable fractures that require surgical fixation to have better clinical outcomes [3].

To our knowledge, our study is the first and largest prospective control study focusing on the best entry points, screw trajectories, and avoidance of wound problems of the TIFI in complete transforaminal sacral fractures in addition to postoperative radiological and functional outcomes assessment. TIFI as a minimally invasive method is valid and effective for management of sacral fractures, with short operative time and low intraoperative fluoroscopy exposure and minimal blood loss.

The novel technique is about the modification of the implantation method of iliac screws: using anatomical entry point (medial and caudal to PSIS, rather than the posterior superior iliac spine at high level) with bone resection around entry point for any prominence in addition to musculofascial closure above iliac screws and submuscular insertion of rod that overcome drawbacks of other implantation techniques especially wound irritation and complications. All these factors, in addition to using a single rod technique (rather than double rod) minimize the posterior soft tissue trauma with better skin healing process. In addition, this standardized protocol allows better TIFI insertion in both the sagittal and axial plane to avoid screw malposition.

Biomechanically, single rod TIFI fixation in this type of fracture acts as a stable stiff construct that can withstand higher stresses than iliosacral screws with superior fixation advantages [15]. Moreover, a retrospective clinical study using the same fixation was conducted on more unstable Tile C pelvic fractures revealing a satisfactory outcome [12]. The role of anterior ring fixation is still debatable, calling for more biomechanical and clinical studies to be conducted [3, 12, 15].

In our study, TIFI technique proved to be a rigid fixation in lateral compression cases that were examined under anesthesia for testing the stability of the anterior ring after posterior fixation [10]. Some cases fixed posteriorly by TIFI did not have anterior ring stabilization, but this might need further biomechanical studies. This finding should be analyzed cautiously due to the presence of multiple confounding variables such as age, anterior ring failure pattern and bone quality.

The practice of posterior spinal instrumentation is a rising trend in surgical fixation based on its high degree of flexibility (rod is modifiable and the screw head is polyaxial) and ease of placement. Rod insertion below the muscular layer is less aggressive than plate application or dual rods providing the construct with strong vertical resistance to the axial pressure [1, 4, 11, 12]. Our resecting bony prominence technique from site of entry following the more anatomical entry point (which is more caudal and medial to PSIS) rather than the traditional entry point significantly improved functional outcome and decreased wound complications.

As the anterior in fix or external fixator hold the anterior ring fractures till appropriate healing occurs without disrupting fracture hematoma or having interfragmentary screws fixation, we believe that posterior in fixator can cause the same effect with no need for sacral fracture compression using iliosacral screw with less demand to image intensifier use, beside avoidance of injury of sacral nerve roots.

Regarding our reduction techniques, different maneuvers can be applied easily to displaced sacral fractures whatever the direction of displacement (vertical and /or dorsal displacement with side gaping). Reduction iliac screws can be used as joysticks for reduction and can be applied through PIIS in different safe corridors. Then, using reduction clamps, displacement can be controlled and anatomically reduced without opening the fracture site. In addition, when applying the final transverse rod to the final iliac crews, rods could be bent gently to buttress against the posterior displacement, maintaining the required sacral position.

Regarding operative and intraoperative fluoroscopy time, our study results was in harmony with Saoud et al. [4], Dienstknecht et al. [13], and Wang et al. [14], but was different from Toda et al. [11] who showed a longer operative time. We used a single rod technique that was comparable to Dienstknecht et al. [13] and Younggang et al. [12] while both Saoud et al. [4] and Toda et al. [11] used a double rod fixation technique with no clear clinical difference. Regarding the functional and radiological outcome assessment, our study was in line with Saoud et al. [4], Younggang et al. [12], Dienstknecht et al. [13] and Wang et al. [14] (Table 1).

**Table 1:** Showing other relevant studies comparable to our study

Study	Year/Country	Methodology/No. of cases	Age	Technique	Fracture classification	Time to surgery (days)	Operative time (minute)	Fluoroscopy time (min)	Blood loss (ml)	Preop. Displacement	Follow-up (m)	Matta score	Function outcome score
Toda et al. [13]	2020 Japan	Retrospective TIFI 27	42.2	Double rod 6 cm incision on each side	Tile; B (26 cases), C1 (1 case) Denis I → 11/II → 16 cases	Mean 6.4 (1–15)	Mean; 129 (60–103)	–	Mean; 223 (100–500)	Mean; 10.7 (1–32)	14.6	Excellent 8 Good; 13 Fair; 6	–
Dienstknecht et al. [15]	2011 Germany	Retrospective TIFI 62	36.7	Single rod Incision;4 cm	Tile C1 (46), C2 (11),C3 (10), 51 were sacral fractures. Denis I → 17, → 32 (II), 2 (III)	1–12	Mean; 29	Mean 0.3 (0.1–1)	Mean; 50	Mean; 8.7 (3–15)	37	Anatomic; 45 Residual displacement < 5 mm; 16 *Gap > 5 mm (1)	German Trauma Society Score Excellent (19) Good (16) Fair (25) Poor (2)
Younggang et al. [14]	2018 China	Retrospective TIFI 47	43.2	Single rod Open reduction	Tile class.: C: all Denis I → 3, Denis II → 24 / Denis III → 2 cases	Mean 7 (5–15)	Mean 184 (67–213)	–	Mean 763 (253–1720)	–	21		Majeed: mean 80.2 Excellent (13) Good (30) Fair (4)
Saoud et al. [4]	2018 Egypt	Prospective TIFI 50	–	Double rod Incision 1.5–2.5 cm on each side	Denis classification I → 10 cases II → 37 cases, III → 3	3–10	Mean: 37.7	–	Mean; 43	–	37	Excellent; 15 Good; 30 Fair; 5	Pohlemann score Excellent(33) good (9) fair (8)
Our study	2022 Egypt	Prospective TIFI 52	30.3 ± 8.7 years	Single rod 1.5–2.5 cm	Tile: (B1:9, B2:52,B3:8 3;C1), Denis: (all cases are type II:)	8.4 (2–21)	Mean: 48.9 (22–65)	Mean: 25.15 s ± 10.4	Mean 312 ml (150–650)	Mean 4.42 (1–18)	18.1 ± 1.6 (12–26)	Mean 3.24 Excellent; 37 Good; 12 Fair: 3	Majeed (1 year) Mean: 88.3 (81–97). Excellent: 42 Good: 10 Mean Pelvic outcome score 33.5 ± 2.6 (29–37)

Some surgeons perform an additional buttressing of the TIFI to the lumbar spine to resist both vertical and horizontal stresses [16, 17]. Patel et al. in his systematic review reported that the most used fixation techniques for unstable ring fractures include iliosacral screws and spinopelvic fixation (SPF). Sagi et al. reported that SPF and triangular osteosynthesis are indicated primarily for the treatment of comminuted transforaminal sacral fractures that are not impacted and have demonstrated vertical instability [17]. However, care needs to be taken for risk of infection and wound complications [16]. Also, biomechanically, our work was comparable to Salasek et al. who compared stiffness between TIFI and ISS in vertically unstable sacral transforaminal fractures. TIFI offers a lower danger of over-compression for the transforaminal fracture line in comparison with ISS [15]. Additional studies are needed to formulate management guidelines for these challenging fractures.

Although the preoperative fracture displacement affects radiological outcome, stable rigid biomechanical construct fixation of sacral fractures could be achieved by TIFI technique. Our modification for transilial screws implantation significantly reduced skin irritation and the necessity for implant removal. This technique allows early mobilization and rehabilitation. Surgeons should be aware of the ideal screw trajectories and entry points for safe successful insertion of the Transilial screws.

Limitations of the study include the short follow-up period, the diversity of the used approaches and methods of fixation for anterior ring injuries or presence of associated fractures. This technique uses posterior implants with a minimally invasive technique; a theoretical disadvantage over ISS screw fixation. The study results are relatively good, when compared to various series describing unstable posterior ring injuries as some cases have minimal displacement and are fixed in situ without reduction, which shows excellent radiological and functional outcome. Comparative randomized studies with larger sample size are needed to compare different techniques of TIFI for unstable pelvic fractures Tile B and C, with longer follow-up period. In addition, single versus double rod TIFI technique in sacral fractures should be compared clinically to confirm the comparable biomechanical strength.

## Conclusion

The novel implantation technique described in our study is associated with minimal wound problems, implant irritation and necessity for implant removal. The results of our study suggest that sacral transforaminal fracture fixation through minimally invasive technique using TIFI give a reliable safe option being an easy technique with short operative time, less radiation exposure than other fixation modalities as well as excellent functional and radiological outcomes regardless different variations in osseous anatomy or dysmorphism. Additional large scale comparative trials should be conducted to validate these results.

## Data Availability

Not applicable; only upon authors request.
